# Stratified dynamic analysis reveals postoperative recovery trajectories of sacral neuromodulation in pediatric neurogenic bladder

**DOI:** 10.3389/fped.2025.1582311

**Published:** 2025-10-09

**Authors:** Yiming Ding, Dongming Wang, Pengxiang Wang, Fangzheng Cheng, Yaru Mou, Yu Zhou, Pengge Xin, Zeyong Niu, Jian Wang

**Affiliations:** ^1^ Department of Pediatric Surgery, Qilu Hospital of Shandong University, Jinan, China; ^2^ Department of Cardiology, Shandong Provincial Hospital Affiliated to Shandong First Medical University, Jinan, China; ^3^ Cheeloo College of Medicine, Shandong University, Jinan, China

**Keywords:** neurogenic bladder, sacral neuromodulation, urodynamic examination, voiding dysfunction, lower urinary tract dysfunction

## Abstract

**Objective:**

To evaluate the effectiveness and safety of sacral neuromodulation (SNM) under general anaesthesia in the treatment of neurogenic bladder (NB) in children, and to share clinical experience.

**Methods:**

We conducted a retrospective analysis of the clinical data of 61 children with neurogenic bladder (NB) who successfully underwent sacral neuromodulation (SNM) treatment at Qilu Hospital of Shandong University from January 2021 to January 2025, with mid-term follow-up (up to 3 years). By tracking key indicators (such as symptomatology and urodynamic parameters) over an extended period, we assessed the safety and efficacy of SNM under general anesthesia for the treatment of pediatric NB and identified general patterns of postoperative recovery.

**Results:**

Preoperatively, all 61 children exhibited symptoms of urinary frequency, urgency, and incontinence. After permanent SNM implantation, compared to baseline, symptoms of voiding dysfunction, including urinary frequency, incontinence, and urinary retention, were significantly alleviated. Urodynamic parameters, including detrusor pressure during the storage phase, bladder compliance, and maximum bladder capacity, also showed statistically significant improvements (*P* < 0.05). Through mid-term follow-up, we modeled postoperative recovery curves using mathematical methods such as restricted cubic splines, and sensitivity analyses confirmed their reliability. Based on these models, subgroup analysis further revealed that the recovery of urinary retention symptoms exhibited different patterns across subgroups.

**Conclusion:**

Sacral neuromodulation (SNM) under general anesthesia is a safe and reliable treatment for pediatric patients. Both early and mid-term postoperative care play a crucial role in optimizing recovery and ensuring sustained therapeutic efficacy.

## 1 Introduction

The prevalence of voiding dysfunction in school-age children is approximately 20% (1, 2), with neurogenic bladder (NB) being one of the major underlying causes. NB is a type of lower urinary tract dysfunction in children that results from lesions in the neuromodulatory system. It is frequently associated with both voiding and bowel dysfunction. When intravesical pressure remains elevated, it often leads to progressive upper urinary tract damage, including vesicoureteral reflux (VUR) and hydronephrosis, and in severe cases, renal failure may occur.

Sacral neuromodulation (SNM) is a safe and feasible treatment for lower urinary tract and bowel dysfunction (3, 4), offering significant improvement in symptoms such as urinary incontinence, urgency, and urinary retention. In pediatric patients, 61%−73% of individuals experience over 50% improvement in these symptoms following SNM treatment (5, 6). However, due to the physical and anatomical differences in children (7, 8), as well as variations in anaesthesia protocols, SNM is predominantly used in adults, with limited application in the pediatric population (9, 10).

The prognosis of neurogenic bladder (NB) in children is markedly worse than in adults. Without treatment, nearly all pediatric patients develop renal failure, with up to 20% mortality in the first year of life (5, 11), whereas in adults the 45-year cumulative risk of severe renal deterioration is about 29% (12). Early intervention is therefore critical in pediatric NB, and the application of SNM offers a promising treatment option (13). Additionally, the development of pediatric-specific surgical approaches is essential (6, 14).

Although the mechanism of SNM is not yet fully elucidated, it is understood that normal urination depends on the coordinated activity of the detrusor and sphincter muscles, regulated by the peripheral ganglia, spinal cord, and brain. Disruption of this neural circuit can lead to loss of reflex control and the development of NB (15).

Current studies suggest that the therapeutic mechanism of electrical stimulation primarily involves modulation of bladder afferent signaling and spinal reflex activity, thereby suppressing detrusor overactivity and improving bladder function (16, 17). Peripheral mechanisms may also include enhanced ATP release and reduced NO release from the urothelium (18), contributing to the maintenance of normal peripheral bladder function (19).

## 2 Information and methods

### 2.1 General information

A total of 61 pediatric patients were enrolled in this study, including 28 males and 33 females, with ages ranging from 6 to 17 years (mean age: 11.6 years). The duration of disease ranged from 1.5 to 14 years.

Based on the classification of spina bifida into open and closed types, 11 children had open spina bifida, all of which were myelomeningocele, while 50 had closed spinal dysraphism, including 27 cases of filum terminale tethering/tethered cord syndrome and 23 cases of lipomyelomeningocele. All closed lesions were confirmed by MRI.

All patients had previously undergone neurosurgical intervention. Children with open spina bifida underwent sac repair during the neonatal period, while those with closed lesions received surgical untethering or lipoma resection either after the onset of symptoms or prophylactically to prevent tethering-related dysfunction.

In the current study, we did not classify patients strictly according to the open/closed anatomical distinction. Instead, we grouped myelomeningocele and lipomyelomeningocele into the congenital malformation group, as both arise from early failure of neural tube closure and are typically associated with bladder dysfunction present at birth. Conversely, tethered cord syndrome was classified into the acquired traction group, as it represents a secondary mechanical pathology where neurological impairment often emerges progressively with growth.

This classification approach minimizes meaningless subgrouping and facilitates a more meaningful comparison of SNM efficacy between two distinct pathophysiological mechanisms. (Detailed patient data were de-identified and are presented in Supplementary Table S1).

### 2.2 Treatment

#### 2.2.1 Primary disease management

In most children with filum terminale tethering, the underlying lesion was still present at diagnosis. In such cases, pediatric neurosurgeons were consulted for joint evaluation. The primary tethering pathology was treated first, and SNM was performed only after postoperative neurological evaluation confirmed surgical candidacy.

#### 2.2.2 Routine evaluation

A detailed medical history and physical examination were performed. Preoperative voiding conditions were recorded continuously over 7 days, including 24 h voiding frequency, voided volume per micturition, Neurogenic Bladder Symptom Score (NBSS), and post-void residual urine volume (measured by catheterization) in children with urinary retention.

#### 2.2.3 Medication and bladder management

A total of 58 patients had received anticholinergic medications and practiced daily clean intermittent catheterization (CIC) prior to surgery.

#### 2.2.4 Laboratory testing

Routine preoperative tests included complete blood count, coagulation profile, urinalysis, serum biochemistry, and urine culture. Serum creatinine and estimated glomerular filtration rate (eGFR) were evaluated for all patients, and results were cross-validated with bilateral renal parenchymal thickness measured by ultrasound. If ultrasound suggested risk of vesicoureteral reflux (VUR), renal parenchymal thinning, or eGFR below the age-specific reference range, additional ^99mTc-DMSA renal scintigraphy was performed to assess differential renal function and exclude upper urinary tract damage.

#### 2.2.5 Imaging and supplementary studies

**Figure 1:**
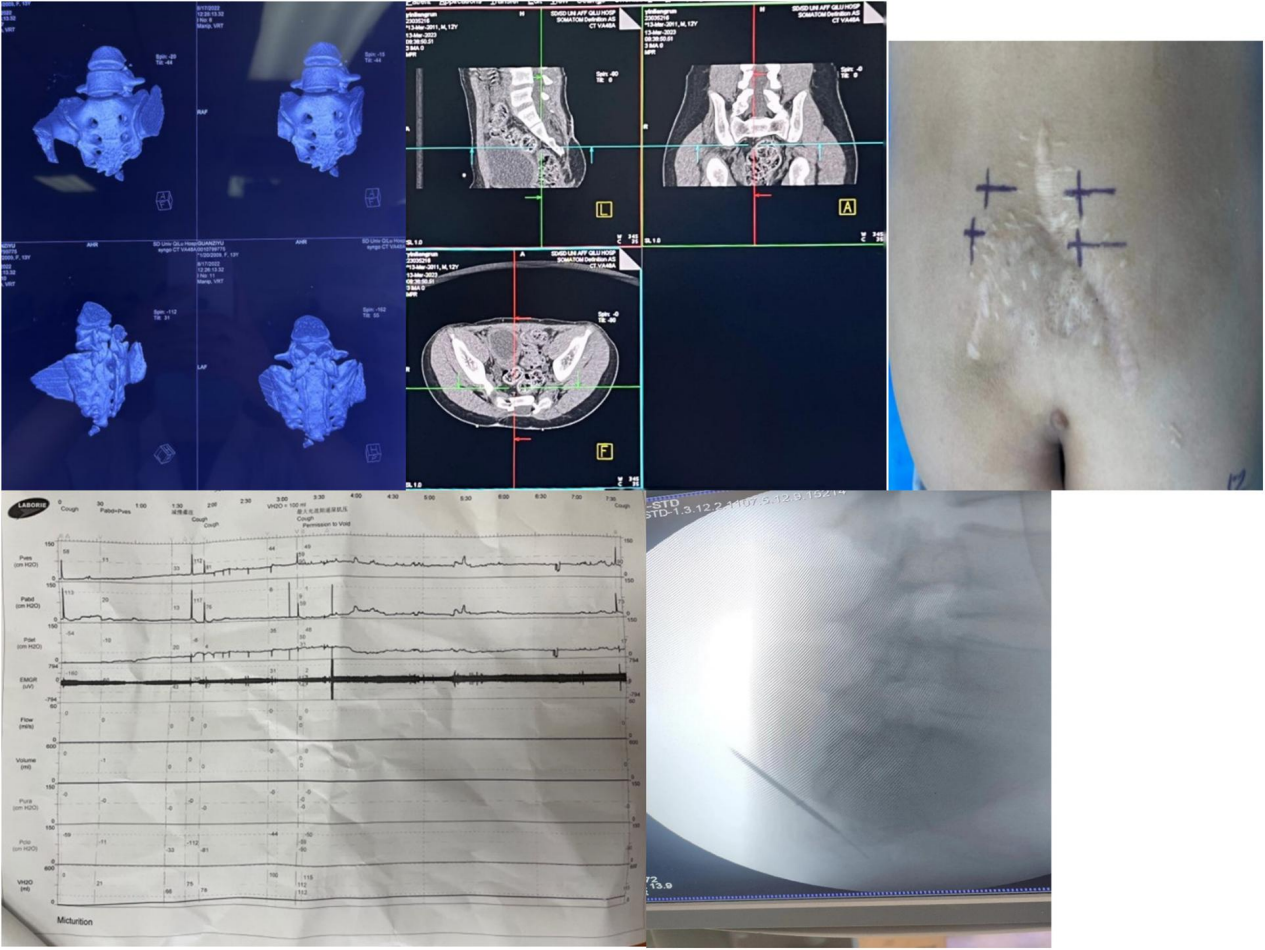
Preoperative localization, baseline urodynamic assessment, and intraoperative electrode placement for sacral neuromodulation (SNM). **(a)** Preoperative sacrococcygeal three-dimensional reconstruction is performed to identify bony deformities and exclude surgical contraindications. **(b)** Computed tomography (CT)–based localization of the S3 foramen facilitates accurate puncture and reduces operative time. **(c)** The body surface puncture site is marked according to CT landmarks, and alternative puncture sites (e.g., contralateral S3, S4, or S2) are prepared in case of inadequate response at S3. **(d)** Baseline urodynamic testing is conducted to evaluate bladder function prior to implantation. **(e)** Intraoperative C-arm fluoroscopy confirms needle entry into the S3 foramen, guides puncture depth, and assists in optimal electrode positioning while ensuring procedural safety.

Preoperative examinations included ECG, chest x-ray, renal ultrasound, and assessment of post-void residual urine. All patients underwent 3D sacral reconstruction (Figure 1), and CT-guided localization of the S3 foramen was performed prior to surgery. In cases where sacral agenesis was identified, 3D simulated puncture localization was performed. (Due to privacy concerns expressed by patients and families, imaging data for sacral agenesis cases are not publicly disclosed.) Informed consent was obtained from all patients and their legal guardians.

#### 2.2.6 Urodynamic testing and additional studies

Conventional urodynamics was first used to establish a baseline of bladder function. If low-pressure VUR was suspected, imaging urodynamics was conducted. If clinical history, imaging, or physical findings suggested anatomical abnormalities, a micturating cystourethrogram (MCUG) was added. All enrollment dates and individualized testing plans have been de-identified and are listed in Supplementary Table S1.

### 2.3 Surgery

Sacral neuromodulation (SNM) was performed in two stages: an experimental treatment period followed by permanent implantation.

#### 2.3.1 First-Stage surgery

The position and depth of the S3 nerve foramen were confirmed preoperatively using CT localization. Following the induction of general anesthesia, the sacrococcygeal region and anus were exposed with the patient in the prone position. The buttocks were secured laterally using adhesive elastic bandages to ensure adequate exposure of the anus for intraoperative observation of neuromuscular responses. Routine surgical sterilization was performed.

Under C-arm fluoroscopic guidance, the S3 neural foramen was punctured, and electrical stimulation was applied. The parameters of the extracorporeal electrical stimulation device were adjusted based on the child's nerve response on either the left or right side.A schematic illustration of the anatomical relationship between the sacrum and the electrode is provided in Supplementary Figure S1. A positive motor response was defined as a bellows-like contraction of the anus or a toe-flexion reflex, without significant contraction of the gastrocnemius muscle. If no response was detected bilaterally at S3, puncture was redirected to the S2 or S4 foramen. If neither S3 nerve foramen elicited a positive response, S2 or S4 was selected as an alternative. If no positive response was observed from these alternative sites, the needle was withdrawn promptly. A positive nerve response was defined as a bellows-like contraction of the anus or an evident toe-flexion reflex, with no significant contraction of the gastrocnemius muscle.

Upon confirming a positive response, a temporary stimulator was implanted. A 3 cm incision was made on the buttock ipsilateral to the electrode site. Following complete separation of the subcutaneous tissue, a subcutaneous tunnel was established, and the electrode wire was positioned appropriately before being externalized through the skin. The wound was thoroughly irrigated and closed with layered suturing. Postoperatively, the stimulation parameters were adjusted according to the child's neuromuscular responses.

All 61 patients underwent successful unilateral S3 puncture. Stimulation parameters were fine-tuned based on postoperative symptom changes. The experimental treatment period lasted for 14 days. Treatment success was defined as an improvement of more than 50% in daily voiding frequency, residual urine volume, and urgency scores compared to baseline. Progression to the second-stage surgery was determined based on the success of the experimental treatment phase and the subjective assessment of both the child and their guardian.

#### 2.3.2 Second-stage surgery

The second-stage surgery followed the same anesthesia and sterilization protocol as the first stage. The original incision was reopened to carefully separate the subcutaneous tissue and muscle while avoiding damage to the implanted electrodes. The previous connection was identified, and the electrodes were carefully detached from the temporary stimulator. The extension wire was removed, and the incision was expanded to accommodate the implantation of the permanent pulse generator. The generator was connected to the existing electrode interface from the experimental phase. After confirming normal functionality, the incision was closed in layers.

### 2.4 Follow-up and trend analysis

All 61 children were followed using a standardized schedule: follow-up visits were conducted at 7 days (week of discharge) and 1 month after the second-stage implantation, followed by monthly outpatient visits. Data were aggregated at six fixed statistical timepoints: 3 months, 6 months, 9 months, 1 year, 2 years, and 3 years postoperatively (maximum follow-up: 3 years).

At each follow-up, patients completed a 7-day voiding diary, recording urinary frequency, retention, and incontinence symptoms, along with NBSS scoring. Residual urine volume was assessed via ultrasound during clinic visits. Stimulator parameters and complications were routinely checked and adjusted as needed.

Objective urodynamic studies were conducted at baseline, 3 months, 6 months, 1 year, 2 years, and 3 years. Monthly urodynamic testing was reserved for patients exhibiting symptomatic fluctuations, to balance clinical burden and data integrity.

This stratified follow-up design allowed for early detection of electrode issues or insufficient stimulation, while providing standardized longitudinal data to assess the sustained efficacy and safety of SNM.

### 2.5 Data integration and processing

#### 2.5.1 Data quality control and indicator selection

The patients in this study were aged 6–17 years. For younger children, the NBSS was often completed with assistance from caregivers, making the direct use of NBSS scores potentially biased in statistical comparisons. Therefore, we selected three objective indicators from the voiding diary—24 h voiding frequency, post-void residual urine volume, and urinary incontinence episodes—as the primary symptom-based variables. The NBSS was used only for consistency verification and exclusion of low-quality records, following these specific criteria:

a. Incontinence frequency: The number of incontinence episodes recorded in the 7-day diary was converted to an estimated monthly frequency and mapped to the NBSS five-grade frequency scale. If the diary grade differed from the NBSS grade by ≥2 levels, it was marked as inconsistent.
b. 24-hour voiding frequency: Based on NBSS Q12 (maximum interval between voids), a theoretical range of daily voids was inferred. Inconsistency with the actual diary-recorded range was marked as conflict.
c. Residual urine: If post-void residual urine >100 ml was recorded in ≥2 catheterizations in one day, the diary entry was labeled “frequent/always incomplete voiding”; otherwise, it was labeled “occasional/never.” If this contradicted NBSS Q15 frequency, the record was flagged.

If any of these conflicts were triggered, the corresponding diary entry was marked as low quality and excluded. Remaining data were used in the longitudinal analysis to ensure the reliability of symptom reporting.

Urodynamic assessment focused on three key parameters: bladder compliance, detrusor pressure during the storage phase, and maximum bladder capacity, reflecting intrinsic compliance, upper urinary tract risk, and bladder storage function, respectively.

Although the primary focus of this study was on urinary symptoms and urodynamic outcomes, bowel function was also descriptively assessed using a simplified questionnaire and diary. Preoperative and 1-month postoperative defecation patterns were classified as constipation (defined as <3 spontaneous bowel movements per week with straining or hard stools) or fecal incontinence (defined as involuntary bowel movements). This aimed to provide an intuitive overview of SNM's effect on bowel symptoms.

#### 2.5.2 Missing data imputation and quality evaluation

First, missing units in both long-format datasets (symptom-based and urodynamic) were identified. Summary statistics showed that the overall missing rate for symptom data was approximately 14% (IQR: 8%–18%), and for urodynamic data, approximately 12% (IQR: 7%–16%). Only a few later follow-up points (up to 3 years) had slightly higher missingness.

We used predictive mean matching (PMM) with chained equations to generate five multiply imputed datasets, which were then combined using Rubin's rules. After imputation, stratified comparisons by “symptom/parameter × follow-up timepoint” were conducted between pre- and post-imputation datasets. We compared:

a. Mean and standard deviation
b. Kolmogorov–Smirnov (KS) distribution tests

Effectiveness of imputation was assessed using the percentage of absolute mean difference relative to original SD and the KS test *p*-value. Overlaid density plots (Supplementary Figure S2) were used to visualize and qualitatively evaluate data consistency in both symptom and urodynamic domains.

**Figure 2:**
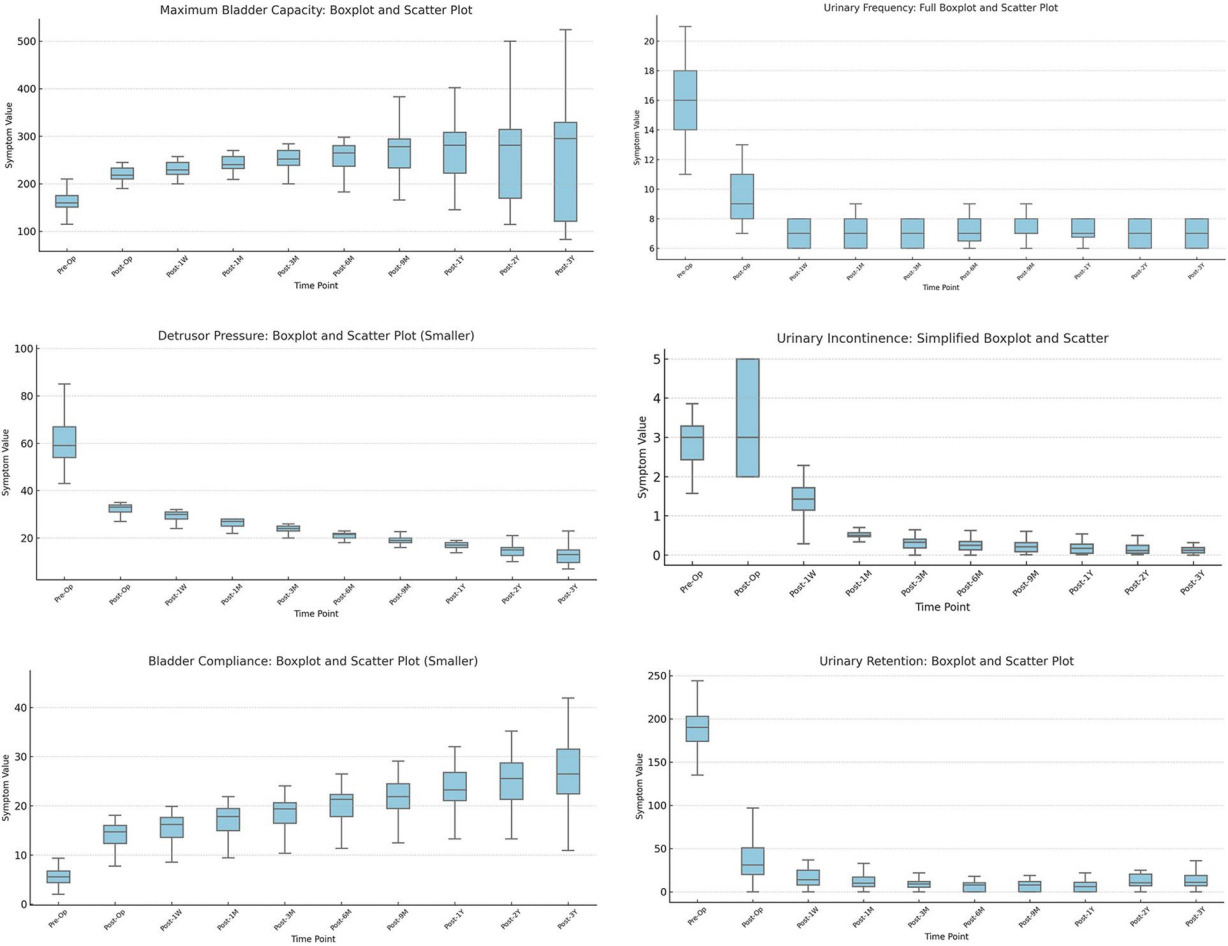
Longitudinal changes in urodynamic parameters and symptom indicators after sacral neuromodulation (SNM). **(a)** Maximum bladder capacity increased after surgery, remaining stable for 9 months before variability widened. **(b)** Detrusor pressure during storage decreased markedly in most children. **(c)** Bladder compliance improved significantly (*P* < 0.01), showing rapid early and slower long-term recovery. **(d)** Urinary frequency decreased substantially and remained stable. **(e)** Urinary incontinence episodes were significantly reduced and nearly eliminated at follow-up. **(f)** Post-void residual urine declined after surgery, though variability persisted.

**Figure 3:**
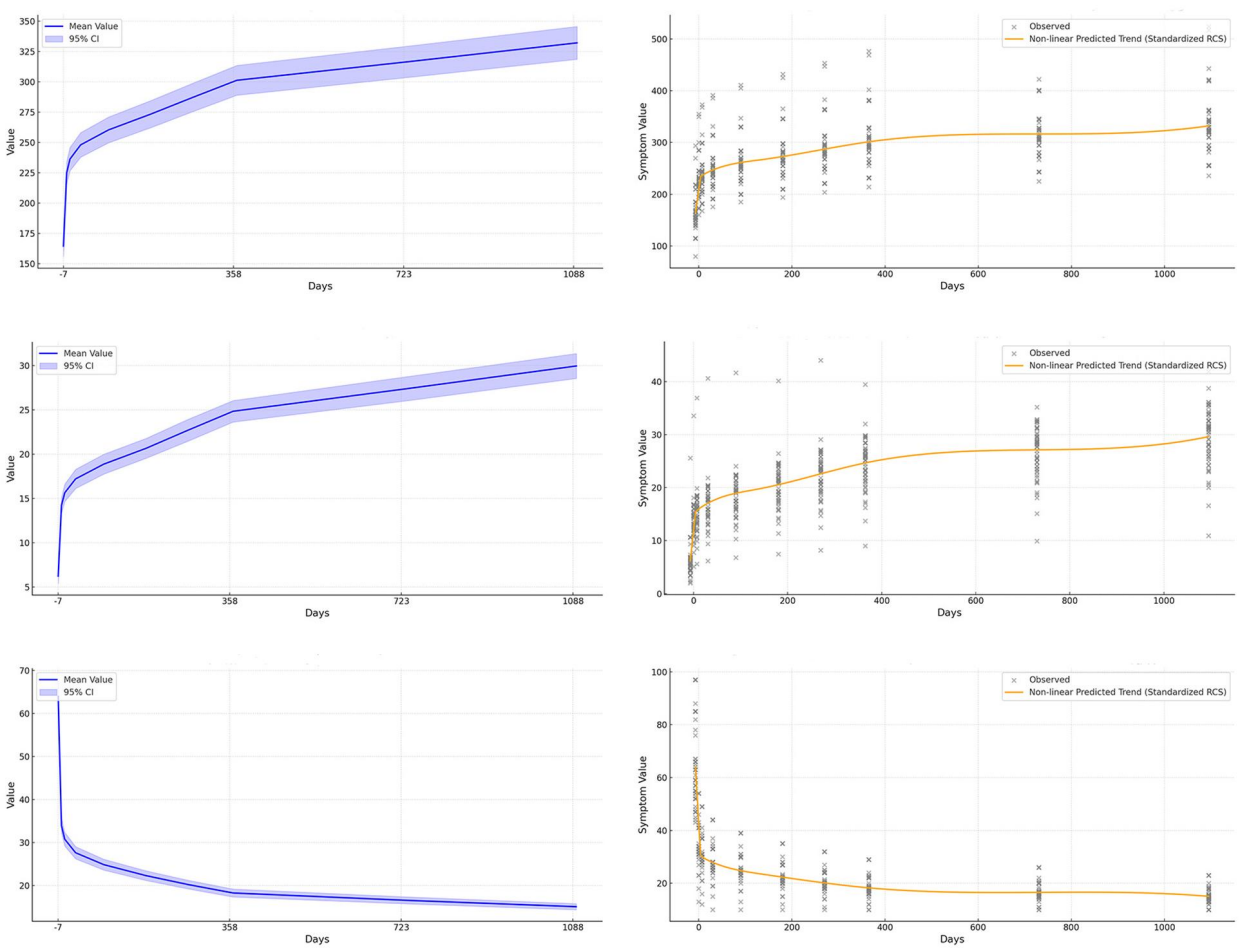
Nonlinear modeling of urodynamic indicators after sacral neuromodulation (SNM). The figure is arranged in two columns: the first column **(a–c)** shows trends and confidence intervals for maximum bladder capacity, bladder compliance, and detrusor pressure during the storage phase, while the second column **(d–f)** applies restricted cubic splines to capture finer nonlinear relationships between postoperative time and changes in these indicators. **(a)** Maximum bladder capacity increases over time with widening variability. **(b)** Bladder compliance shows steady improvement with extended follow-up. **(c)** Detrusor pressure during storage decreases significantly and remains low. **(d)** Restricted cubic spline modeling of maximum bladder capacity reveals nonlinear long-term recovery trends. **(e)** Restricted cubic spline modeling of bladder compliance highlights both rapid early and gradual long-term improvement. **(f)** Restricted cubic spline modeling of detrusor pressure captures nonlinear decline and stabilization patterns.

**Figure 4:**
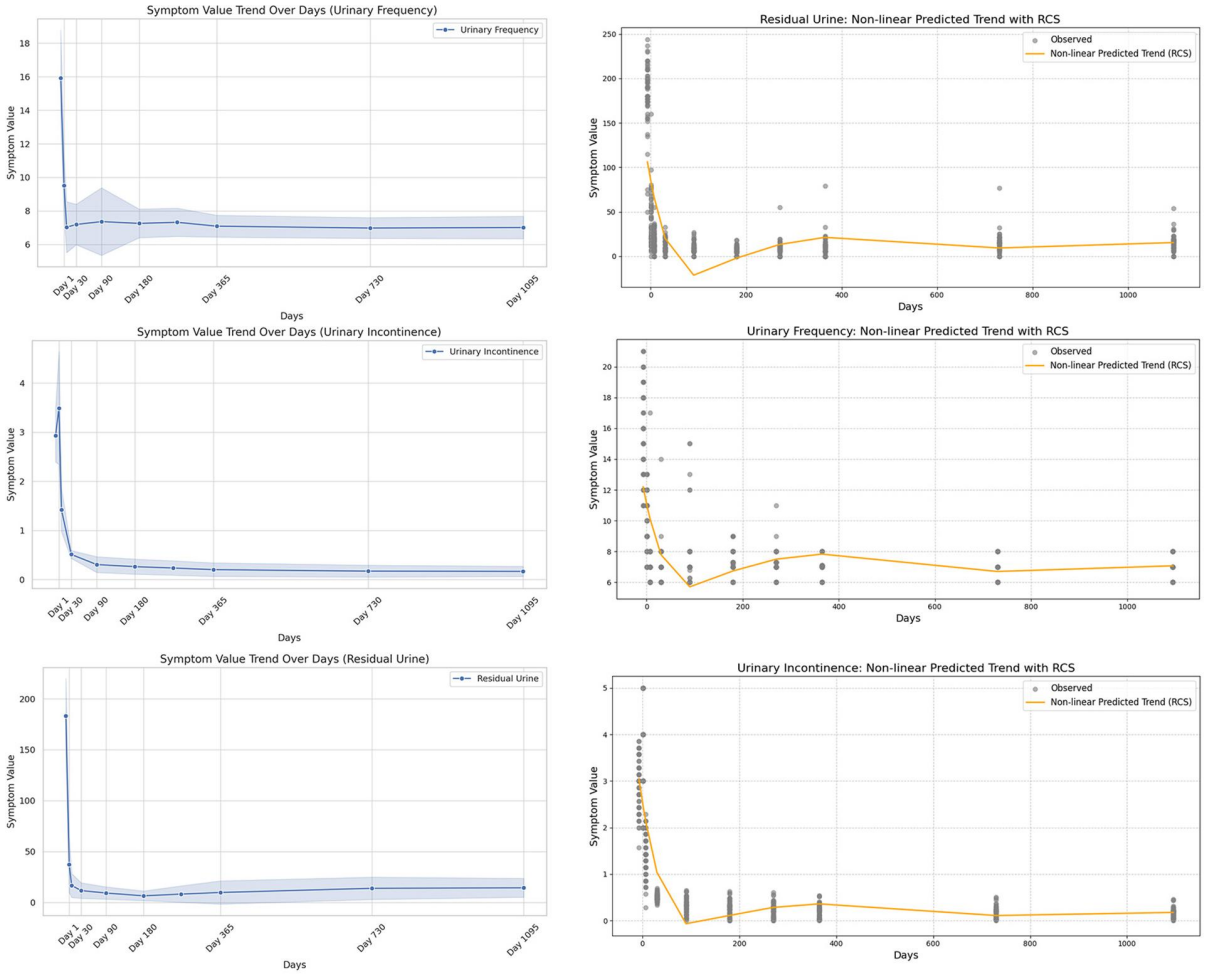
Nonlinear modeling of symptom indicators after sacral neuromodulation (SNM). The left column **(a–c)** shows trends in urinary frequency, urinary incontinence, and urinary retention; the right column **(d–f)** applies restricted cubic splines (RCS) to capture detailed nonlinear changes. **(a)** Urinary frequency shows a steady decline after surgery. **(b)** Urinary incontinence decreases markedly during follow-up. **(c)** Urinary retention gradually improves with time. **(d)** RCS modeling reveals nonlinear decline of urinary frequency. **(e)** RCS modeling illustrates nonlinear reduction of incontinence episodes. **(f)** RCS modeling shows nonlinear recovery pattern of urinary retention.

Finally, we used the imputed complete dataset to visualize the distributions of six key parameters using box plots (Figure 2; Tables 1, 2), and displayed time-based trends with line graphs. Restricted cubic splines (RCS) were used to model postoperative recovery trajectories (Figures 3, 4).

**Table 1:** Comparison of lower urinary tract symptoms in patients before and after SNM.

Urination	Number of examples	Preoperative assessment	Post-operative assessment	Statistical value
24 h urination (times/day)	61	15.92 ± 2.87	7.03 ± 1.52	T = 21.57 *P* = 0.000
Residual urine volume (ml)	61	183.13 ± 36.68	58.48 ± 30.09	T = 33.75 *P* = 0.000
Urinary Incontinence	61	2.93 ± 0.54	1.42 ± 0.45	T = 16.90 *P* = 0.000

**Table 2:** Comparison of urodynamic parameters before and after SNM.

Urodynamic parameters	Number of examples	Preoperative assessment	Post-operative assessment	Statistical value
Bladder compliance (ml/cmH2O)	61	6.22 ± 3.31	15.63 ± 3.90	T = −14.35 *P* = 0.000
Storage phase detrusor pressure (cm/H2O)	61	64.02 ± 15.36	30.75 ± 6.35	T = 15.63 *P* = 0.000
Maximum filling volume (ml)	61	164.59 ± 34.70	236.30 ± 38.57	T = −10.79 *P* = 0.000

### 2.6 Bias control and elimination

**Figure 5:**
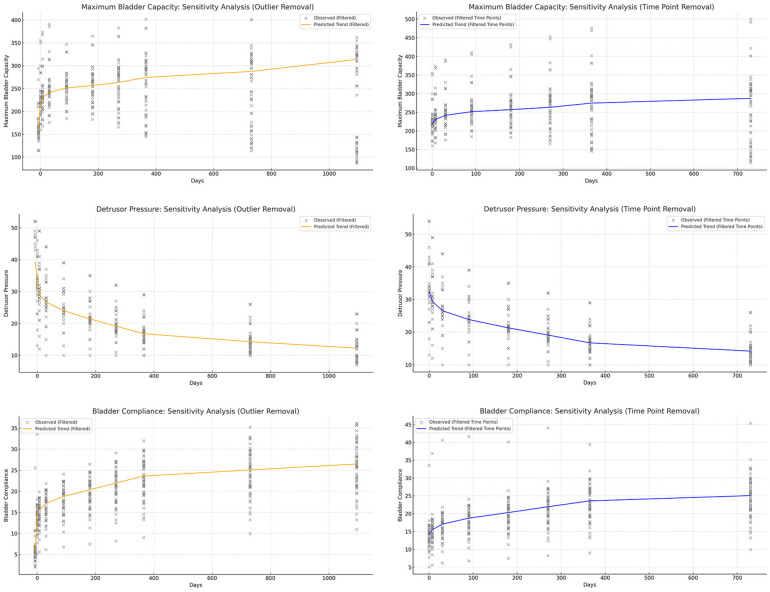
Sensitivity analysis of urodynamic parameters, showing that the model remains robust after removing outliers and excluding data at 30 and 365 days. **(a)** Maximum bladder capacity trends remained stable after removing extreme values. **(b)** Detrusor pressure patterns were consistent with the original model without dependence on specific time points. **(c)** Bladder compliance showed similar improvement trajectories, unaffected by outliers. **(d)** Urinary frequency trends were preserved after sensitivity adjustments. **(e)** Urinary incontinence outcomes remained robust following data exclusion. **(f)** Post-void residual urine analysis demonstrated stability regardless of excluded intervals.

**Figure 6:**
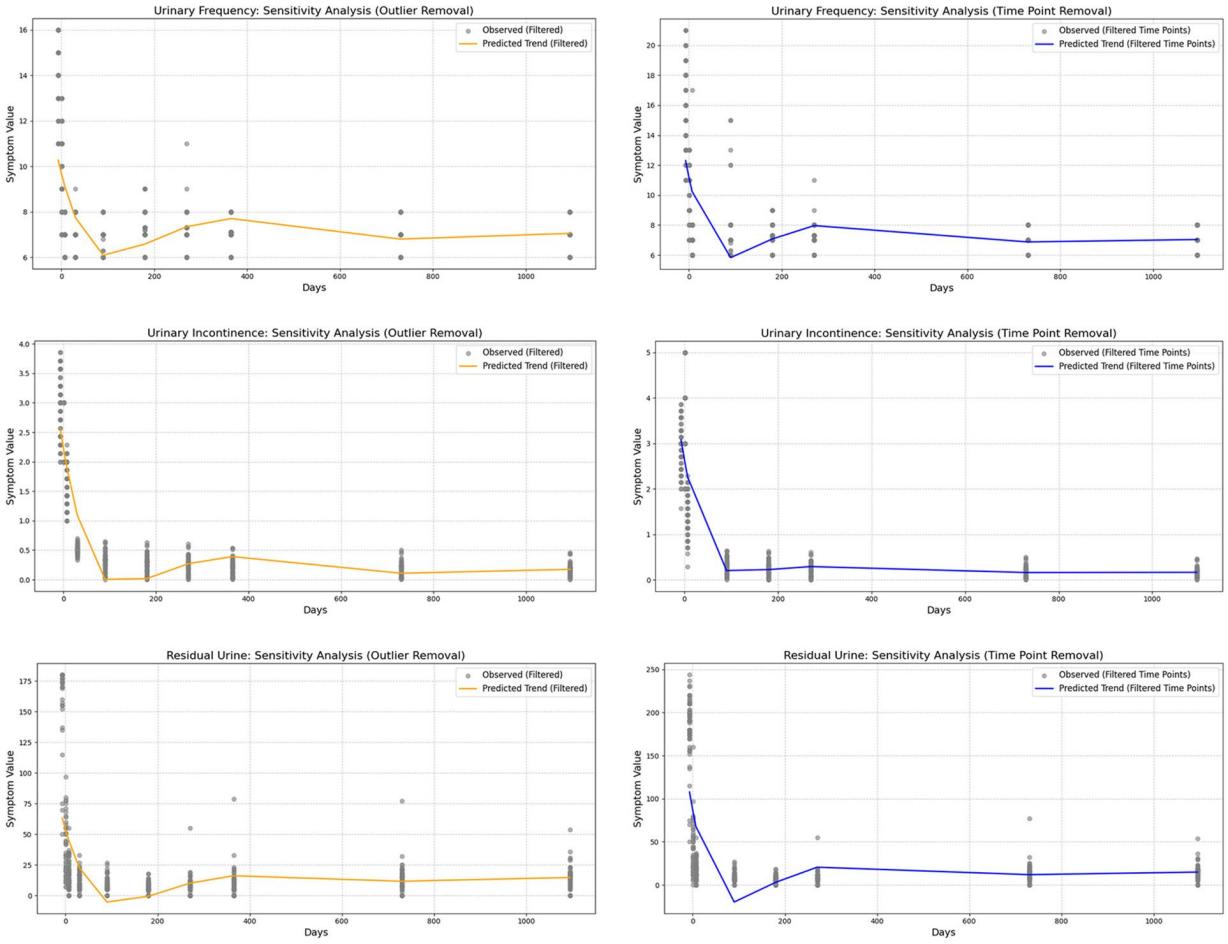
Sensitivity analysis of symptom indicators, showing that the predictive model remains robust and is not dependent on extreme values or specific time points.

To minimize the influence of specific values on the conclusions, we applied a sensitivity analysis by refitting the restricted cubic splines (RCS) after removing certain extreme values or specific time points (Figures 5, 6). This approach allowed us to assess whether the postoperative recovery trend curve was overly influenced by outliers or specific data points, ensuring that the observed trends were robust and not driven by isolated values.

In small-sample retrospective studies, controlling for specific value biases helps prevent results from being overly influenced by isolated data points while ensuring that the overall trend remains accurately represented. By applying this method, we enhance the robustness and reliability of our conclusions, minimizing the risk of drawing misleading inferences due to disproportionate weighting of certain values.

### 2.7 Comparison of short-term and mid-term postoperative follow-up

**Figure 7:**
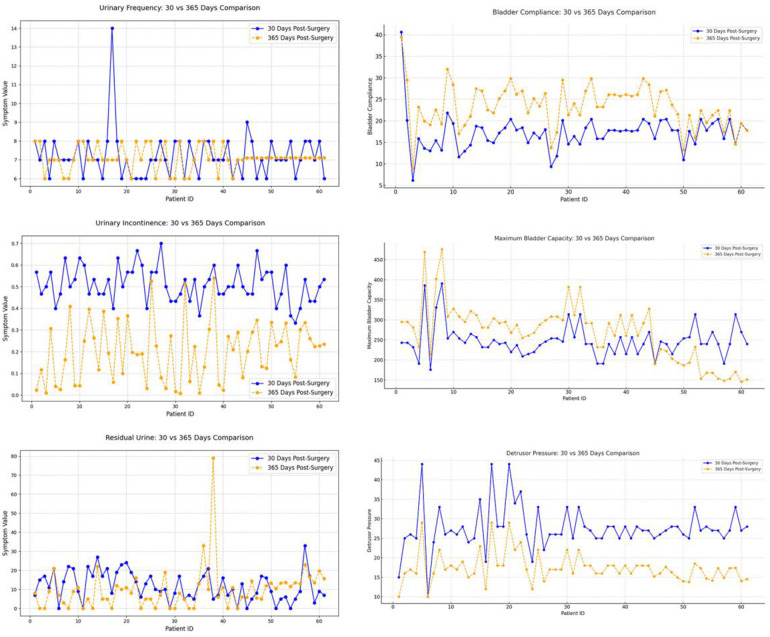
Six major bladder function indicators at 30 and 365 days postoperatively: urinary frequency (upper left), bladder compliance (upper right), urinary incontinence (middle left), maximum bladder capacity (middle right), residual urine (lower left), and detrusor pressure (lower right). Marked improvements were observed in urinary incontinence, bladder compliance, and detrusor pressure by one year, while changes in urinary frequency and residual urine were less pronounced.

This study conducted a longitudinal comparison of short-term (30 days postoperatively) and mid-term (365 days postoperatively) follow-up data to evaluate postoperative recovery trends (Table 3). Symptomatology indicators (24 h voiding frequency, urinary incontinence frequency, post-void residual urine volume) and urodynamic parameters (bladder compliance, detrusor pressure during the storage phase, maximum bladder capacity) were analyzed using a patient-matched approach to minimize inter-individual variability. Line graphs were used to visualize individual changes at both time points, with distinct colors representing short-term and mid-term data for direct comparison (Figure 7). By assessing the trends and variations over time, this method provides insights into the stability and mid-term efficacy of postoperative recovery, offering clinical implications for patient management.

**Table 3:** Comparison of short-term and Mid-term postoperative follow-Up.

Key indicators	Statistical value
24 h urination (times/day)	*P* = 0.562
Residual urine volume (ml)	*P* = 0.275
Urinary incontinence	*P* = 0.000
Bladder compliance (ml/cmH2O)	*P* = 0.000
Storage phase detrusor pressure (cm/H2O)	*P* = 0.000
Maximum filling volume (ml)	*P* = 0.000

### 2.8 Subgroup analysis of short-term and mid-term postoperative follow-up

**Figure 8:**
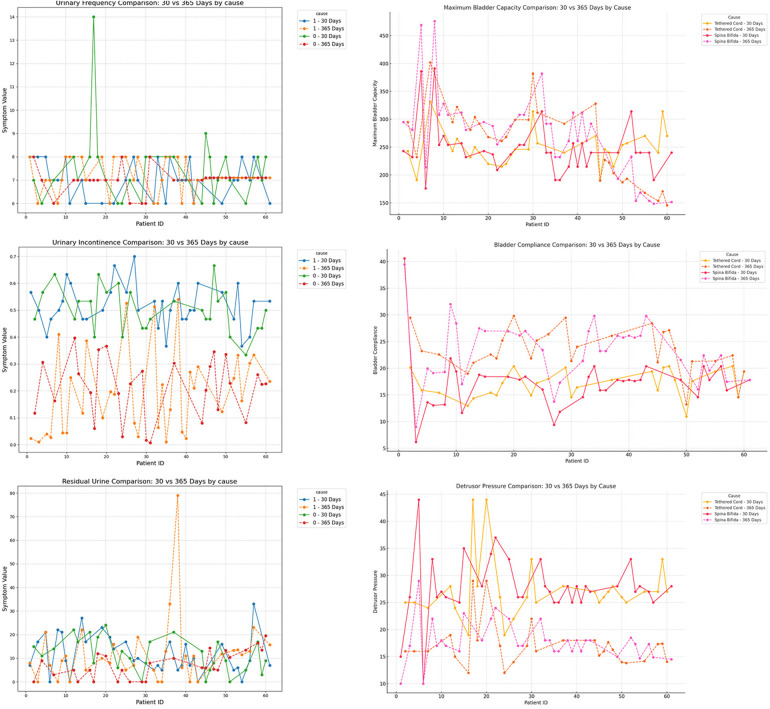
Subgroup analysis by etiology (cause = 0, tethered cord; cause = 1, spina Bifida), comparing urinary frequency, maximum bladder capacity, urinary incontinence, bladder compliance, residual urine, and detrusor pressure at postoperative day 30 and day 365.

**Figure 9:**
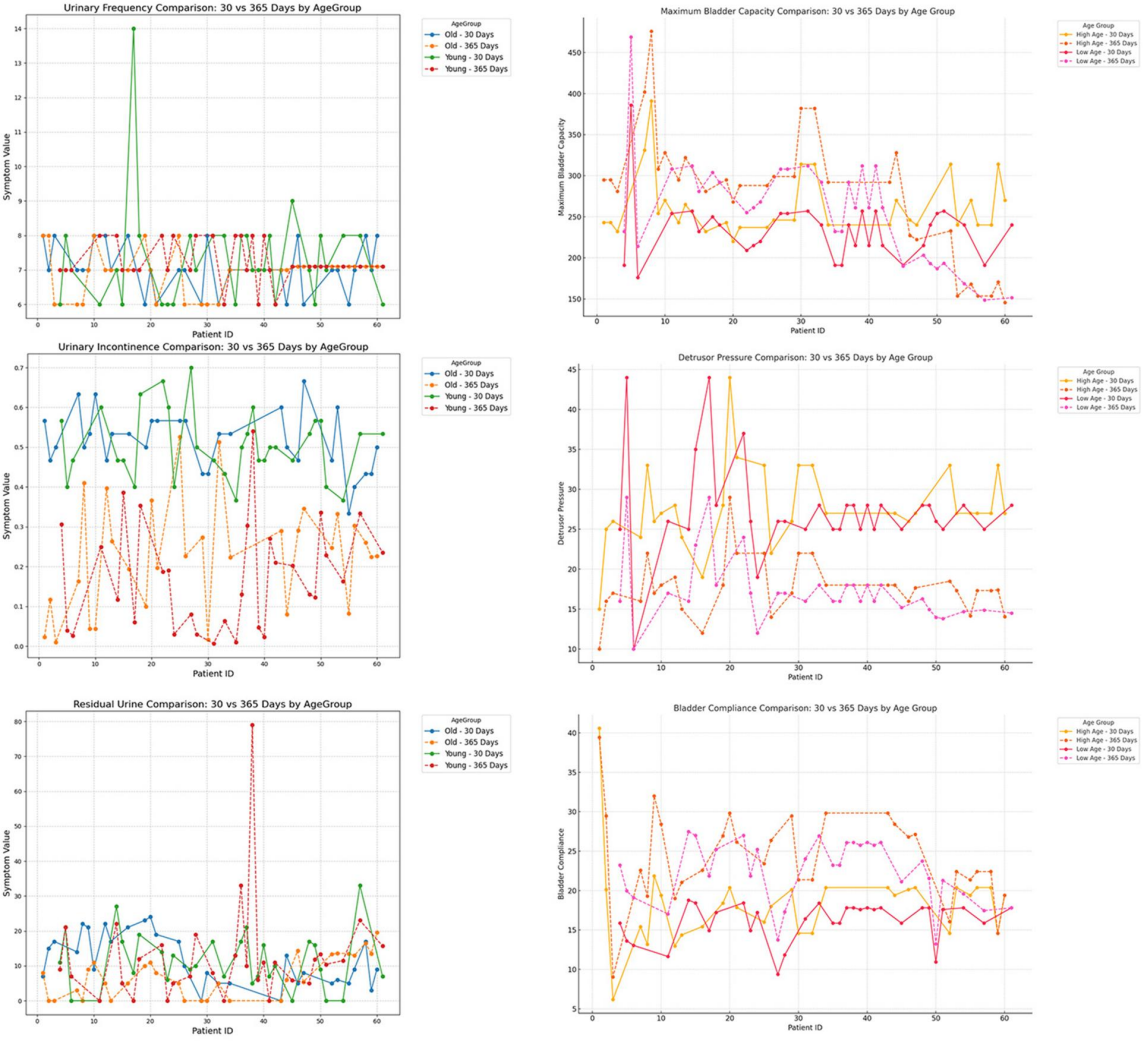
Subgroup analysis by age (<12 years vs. ≥12 years), comparing urinary frequency, maximum bladder capacity, urinary incontinence, detrusor pressure, residual urine, and bladder compliance at postoperative day 30 and day 365.

**Figure 10:**
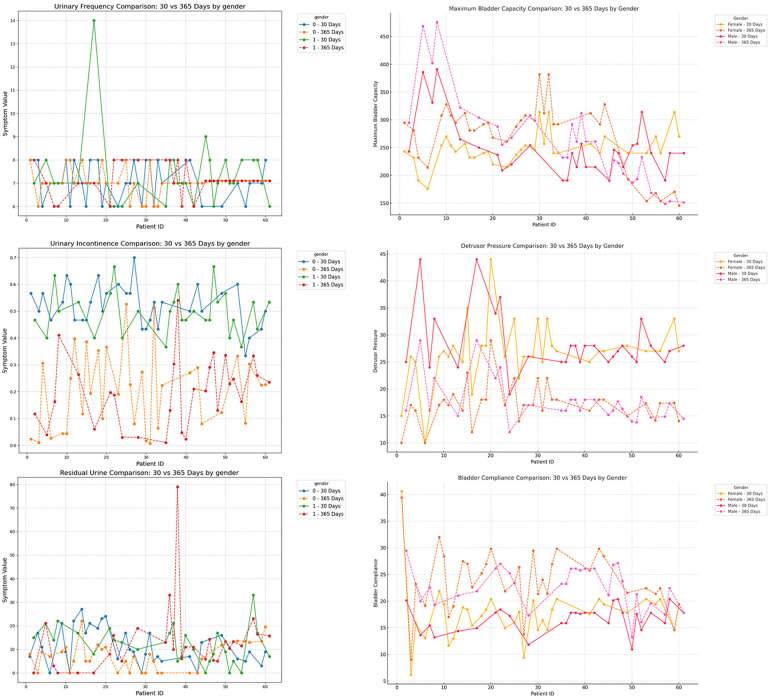
Subgroup analysis by gender (male vs. female), comparing urinary frequency, maximum bladder capacity, urinary incontinence, detrusor pressure, residual urine, and bladder compliance at postoperative day 30 and day 365.

To further investigate the impact of patient characteristics on postoperative recovery trends, a subgroup analysis was conducted based on etiology (tethered cord syndrome vs. spina bifida), age (high vs. low age group), and gender (male vs. female), which were showen in Figures 8–10. Symptomatology indicators (24 h voiding frequency, urinary incontinence frequency, post-void residual urine volume) and urodynamic parameters (bladder compliance, detrusor pressure during the storage phase, maximum bladder capacity) were compared at 30 and 365 days postoperatively, with line graphs used to illustrate individual-level changes over time. This analysis aimed to assess recovery patterns among different subgroups, identify potential heterogeneity, ensure the robustness of conclusions, and provide insights for personalized postoperative management. If certain subgroups exhibited poorer recovery at mid-term follow-up, it may indicate the need for adjusted follow-up schedules or optimized rehabilitation strategies, ultimately enhancing postoperative outcomes and informing clinical decision-making.

### 2.9 Statistical methods

Data were analyzed using GraphPad Prism 8 and python. Continuous variables with a normal distribution were expressed as mean ± standard deviation and compared using paired *t*-tests. Non-normally distributed data were expressed as median (interquartile range), and comparisons were made using the signed rank-sum test.

## 3 Results

### 3.1 Clinical symptoms

The experimental treatment phase lasted 14 days, during which no infections, electrode displacements, or rejection reactions were reported. A total of 65 children received treatment at our institution, of whom 61 successfully proceeded to the second-stage surgery, with all exhibiting a positive response postoperatively (defined as an improvement of more than 50% in at least one of the three symptoms). Four children withdrew due to unsatisfactory efficacy and did not undergo postoperative urodynamic assessment; thus, they were excluded from the study. The differences in all clinical indicators before and after surgery were statistically significant (*p* < 0.05).

### 3.2 Follow up

Postoperative follow-up data demonstrated a continuous improvement in urodynamic parameters, with the most significant gains observed within the first 180 days after surgery. However, during this phase, the parameters had not yet returned to normal levels. Beyond 180 days, patients entered a sustained recovery phase, during which these parameters continued to improve and eventually stabilized.

Symptom-based indicators showed a rapid decline in the early postoperative period and plateaued around one year after surgery, with no noticeable rebound during the follow-up period.

The restricted cubic spline (RCS) regression model revealed that recovery trajectories were not linear but followed a phase-dependent pattern. For example, 24 h voiding frequency exhibited greater fluctuation in the early postoperative period, whereas post-void residual volume showed more variability during the later recovery phase. These trends, reflected by differences in confidence interval widths, suggest individual variability in the timing and pattern of recovery across different indicators. Such findings provide clinical insight into identifying critical time windows for intervention and evaluation.

In the subgroup analysis, several indicators differed significantly among groups. For mid- to long-term postoperative recovery of urinary retention, children in the acquired-traction group (cause = 1) showed greater potential for further improvement than those in the congenital-malformation group (cause = 0) (20) (Table 4a; *P* < 0.05). Similarly, females exhibited a higher recovery potential than males (Table 4b; *P* < 0.05), and the older age group showed greater recovery potential compared to the younger age group (Table 4c; *P* < 0.05).

**Table 4:** Subgroup Analysis (30d vs. 365d).

a		
Key indicators	Subgroup	Statistical value
24 h urination (times/day)	Cause = 1	*P* = 0.379
24 h urination (times/day)	Cause = 0	*P* = 0.173
Residual urine volume (ml)	Cause = 1	*P* = 0.940
Residual urine volume (ml)	Cause = 0	*P* = 0.017
Urinary incontinence	Cause = 1	*P* = 0.000
Urinary incontinence	Cause = 0	*P* = 0.000
Bladder compliance (ml/cmH2O)	Cause = 1	*P* = 0.000
Bladder compliance (ml/cmH2O)	Cause = 0	*P* = 0.000
Storage phase detrusor pressure cm/H2O)	Cause = 1	*P* = 0.000
Storage phase detrusor pressure (cm/H2O)	Cause = 0	*P* = 0.000
Maximum filling volume (ml)	Cause = 1	*P* = 0.000
Maximum filling volume (ml)	Cause = 0	*P* = 0.000
b		
Key indicators	Subgroup (0 = female; 1 = male)	Statistical value
24 h urination (times/day)	Gender = 1	*P* = 0.520
24 h urination (times/day)	Gender = 0	*P* = 0.948
Residual urine volume (ml)	Gender = 1	*P* = 0.776
Residual urine volume (ml)	Gender = 0	*P* = 0.004
Urinary incontinence	Gender = 1	*P* = 0.000
Urinary incontinence	Gender = 0	*P* = 0.000
Bladder compliance (ml/cmH2O)	Gender = 1	*P* = 0.000
Bladder compliance (ml/cmH2O)	Gender = 0	*P* = 0.000
Storage phase detrusor pressure (cm/H2O)	Gender = 1	*P* = 0.000
Storage phase detrusor pressure (cm/H2O)	Gender = 0	*P* = 0.000
Maximum filling volume (ml)	Gender = 1	*P* = 0.000
Maximum filling volume (ml)	Gender = 0	*P* = 0.000
c		
Key indicators	Subgroup of age	Statistical value
24 h urination (times/day)	High age group	*P* = 0.265
24 h urination (times/day)	Low age group	*P* = 0.919
Residual urine volume (ml)	High age group	*P* = 0.002
Residual urine volume (ml)	Low age group	*P* = 0.587
Urinary incontinence	High age group	*P* = 0.000
Urinary incontinence	Low age group	*P* = 0.000
Bladder compliance (ml/cmH2O)	High age group	*P* = 0.000
Bladder compliance (ml/cmH2O)	Low age group	*P* = 0.000
Storage phase detrusor pressure (cm/H2O)	High age group	*P* = 0.000
Storage phase detrusor pressure (cm/H2O)	Low age group	*P* = 0.000
Maximum filling volume (ml)	High age group	*P* = 0.000
Maximum filling volume (ml)	Low age group	*P* = 0.000

Based on preoperative assessment using both questionnaires and defecation diaries, 29 children were diagnosed with bowel dysfunction (including constipation or fecal incontinence). One month after surgery, only 7 children continued to exhibit abnormal bowel symptoms, indicating that SNM treatment led to marked improvement in bowel function for the majority of patients.

### 3.3 Postoperative complications

#### 3.3.1 Short-term complications

During the postoperative recovery period, none of the children experienced symptoms such as infection or bleeding. Within the first year after surgery, some patients (21/61) reported a sensation of stimulation due to electrical current. After parameter adjustments, the majority of these patients (19/21) experienced symptom relief.

#### 3.3.2 Mid-term complications

Two cases of lead breakage were observed. Both patients experienced rapid height growth (>20 cm) within three years after surgery (sitting height changes were not measured). After surgical lead replacement, the therapeutic effects were restored.

## 4 Discussion

### 4.1 Surgical technique improvements

In this study, we lowered the surgical age threshold for sacral neuromodulation (SNM) in pediatric neurogenic bladder (NB) to 6 years, aiming for early intervention before irreversible urinary tract dysfunction occurs. This age is below the reported median of 10.8 years in previous studies (21), reported in previous studies, yet favorable outcomes were still achieved. However, we observed that younger children (6–8 years) often required parental assistance to complete preoperative questionnaires, which introduces potential bias. Therefore, in such cases, clinicians should place greater emphasis on objective data—such as voiding diaries and urodynamic parameters—rather than subjective symptom scores.

Compared to earlier literature (22), our study highlights the importance of general anesthesia in pediatric SNM. While adult SNM procedures rely on patients’ real-time feedback to guide electrode positioning and stimulation, young children often cannot reliably communicate sensations. This necessitates more objective criteria for optimal lead placement and stimulation strength. All procedures in this study were performed under general anesthesia to eliminate errors caused by involuntary movements. Preoperative CT was used for precise localization of the puncture site, combined with intraoperative C-arm fluoroscopic guidance, which significantly improved electrode placement accuracy. Postoperative follow-up demonstrated notable improvement in urodynamic parameters and lower urinary tract symptoms, along with a reduced risk of upper urinary tract damage. Although SNM has proven efficacy in pediatric NB, mid-term follow-up remains essential to evaluate treatment durability and refine surgical strategies for broader clinical application (21, 23).

A key clinical insight from this study is the frequent delay in diagnosis and treatment among children. Early symptoms of defecation and voiding dysfunction are often nonspecific, leading many parents to prolong the use of diapers and delay initial consultations. Moreover, the first medical visit is often to urology rather than neurosurgery, further postponing correct diagnosis and treatment. Even among those who underwent timely untethering procedures after diagnosis of tethered cord syndrome, chronic traction may have already caused irreversible neurological damage, which decompression surgery alone cannot fully reverse. As a result, voiding and defecation disorders may persist, and SNM remains necessary postoperatively. Therefore, earlier identification of tethered cord syndrome and prompt neurosurgical referral may reduce the likelihood of requiring subsequent SNM.

In addition to improvements in urinary symptoms, our results showed a significant reduction in bowel dysfunction: from 29 children preoperatively to only 7 at 1 month postoperatively. This suggests that SNM may also positively impact bowel function. Although bowel symptoms were not deeply analyzed in this study, these preliminary findings support the view that bowel benefit may be an important adjunctive outcome of SNM, aligning with existing clinical evidence (24, 25).

### 4.2 Summary of postoperative recovery patterns

Lower urinary tract symptoms, including urgency, frequency, and incontinence, showed significant improvement shortly after electrode implantation. Among the 61 children, all reported abnormal bladder sensation, with 54 experiencing urgency (UD) upon first sensation of bladder filling and 7 reporting bladder discomfort or pain preceding incontinence. In our cohort, incontinence was generally preceded by urgency or discomfort, indicating that the episodes were more often associated with urgency than with a complete lack of sensation. Among the four patients with unsatisfactory first-stage outcomes, three reported a complete lack of bladder sensation, and they did not perceive the event even when incontinence occurred.

In the postoperative early phase (within 180 days), the confidence interval for 24 h voiding frequency was relatively wide, likely due to individual variability in the recovery rate of bladder sensation (26). In contrast, urinary retention exhibited an increasing confidence interval during mid-term follow-up (beyond 180 days), which may be associated with growth and physiological changes in pediatric patients over time.

For urodynamic parameters, greater emphasis should be placed on long-term recovery (27). While these parameters improved rapidly in the early postoperative phase, this initial recovery may primarily result from the resolution of bladder inflammation and abnormal bladder sensation (28). However, despite this early improvement, urodynamic function remained far from normal levels. The true restoration to normal function occurred during the gradual mid-term recovery phase (up to 3 years). This pattern is further supported by the significant differences (*P* < 0.001) between postoperative day 30 and day 365 urodynamic measurements, highlighting the necessity of long-term follow-up (29).

### 4.3 Device integrity

In this study, two cases of lead breakage were identified. Both incidents occurred in children who experienced rapid overall height growth of more than 20 cm, which we consider the main factor contributing to lead rupture. Although seated height was not directly measured, it is plausible that an associated increase in seated height further amplified the mechanical strain on the leads. Patient interviews also revealed that device malfunction in both cases occurred shortly after large-amplitude movements, such as forward trunk flexion in the seated position and long jumping, suggesting that growth-related and activity-related factors may have acted synergistically to cause lead failure.

Based on these cases, we hypothesize that children undergoing rapid growth may be at higher risk for lead rupture (21, 30), and that intense physical activity—especially involving hip flexion—may further contribute to device damage. These two risk factors may have a cumulative effect, jointly increasing the likelihood of equipment failure. Therefore, for patients experiencing rapid height gains (e.g., >20 cm increase in seated height), activity modification may be warranted. In particular, large-range hip flexion movements should be limited to reduce the risk of mechanical failure.

### 4.4 Limitations and future directions

This study has several limitations that should be acknowledged. As a single-center retrospective analysis with a modest sample size, the generalizability of the findings may be limited. Although missing data at later follow-up points were handled through multiple imputation and validated statistically, prospective studies are necessary to confirm the observed trends. The assessment of bowel symptoms was simplified and lacked detailed stratification or mechanistic interpretation. Additionally, due to a maximum follow-up duration of three years, we were unable to assess mid-term device performance, such as battery longevity or generator replacement, because the maximum follow-up in this study was limited to three years.

Future research should include prospective, multicenter trials with larger cohorts and longer follow-up to evaluate the sustained efficacy and durability of SNM in children. Further exploration of the neural mechanisms underlying SNM's effects on bowel function—particularly its potential modulation of central autonomic pathways—is warranted (31). Moreover, technical innovation is needed to develop pediatric-specific expandable lead systems that can accommodate somatic growth and reduce the risk of device-related mechanical complications.

## 5 Conclusion

Sacral neuromodulation (SNM) under general anesthesia is a safe and reliable treatment for pediatric patients. Both early and mid-term postoperative care play a crucial role in optimizing recovery and ensuring sustained therapeutic efficacy.

## Data Availability

The datasets presented in this article are not readily available because the dataset is available upon request to the corresponding author. However, access to the data may be subject to certain restrictions, including privacy and confidentiality concerns related to patient information. Any requests for data will be evaluated on a case-by-case basis, and shared data will be anonymized to ensure compliance with ethical and privacy standards. Requests to access the datasets should be directed to for dataset access requests, please contact the corresponding author at the following email address: wjdoctor2021@126.com.
