# Supplementary figures and images for: Stratified dynamic analysis reveals postoperative recovery trajectories of sacral neuromodulation in pediatric neurogenic bladder
Source: Front Pediatr. 2025 Oct 9;13:1582311. doi: 10.3389/fped.2025.1582311 (PMC12545125; doi:10.3389/fped.2025.1582311)

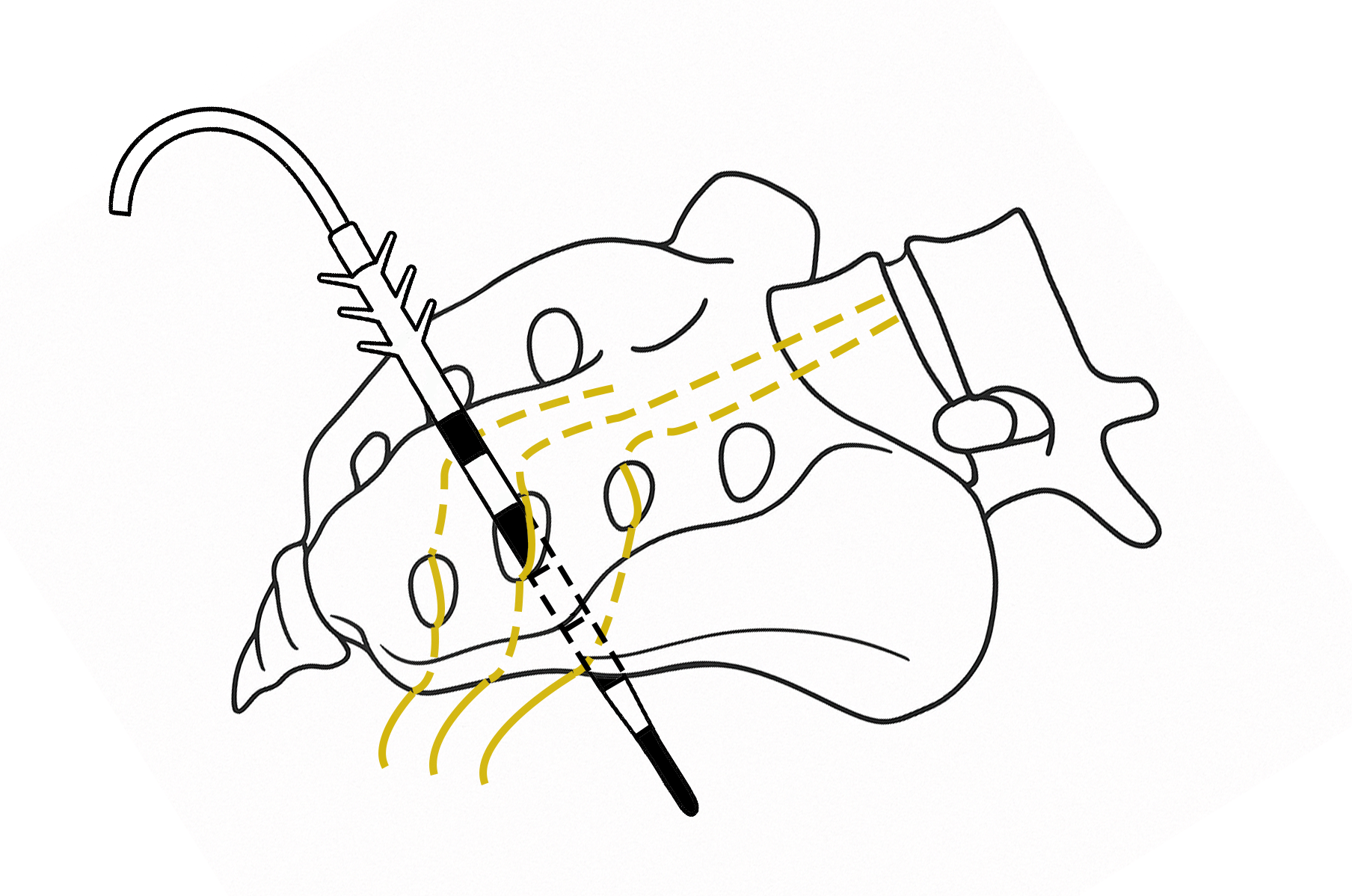

Supplement: Supplementary file 2 [file Image1.png]

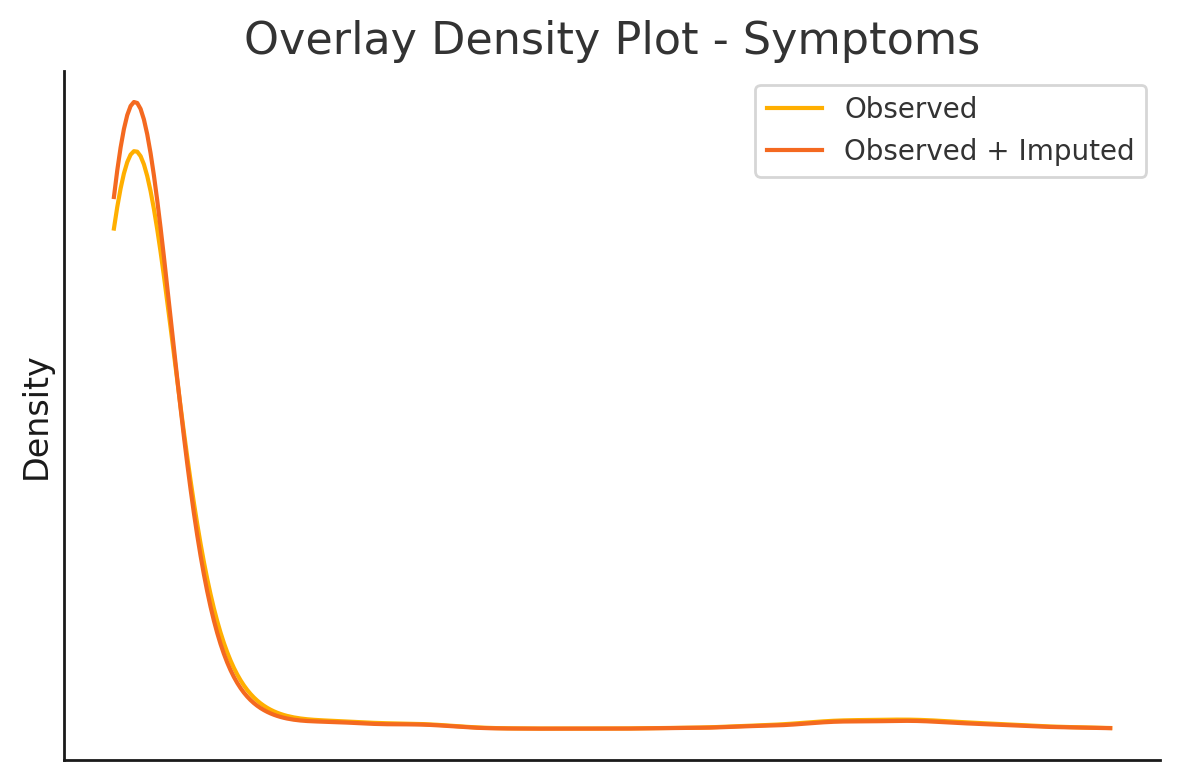

Supplement: Supplementary file 3 [file Image2.png]

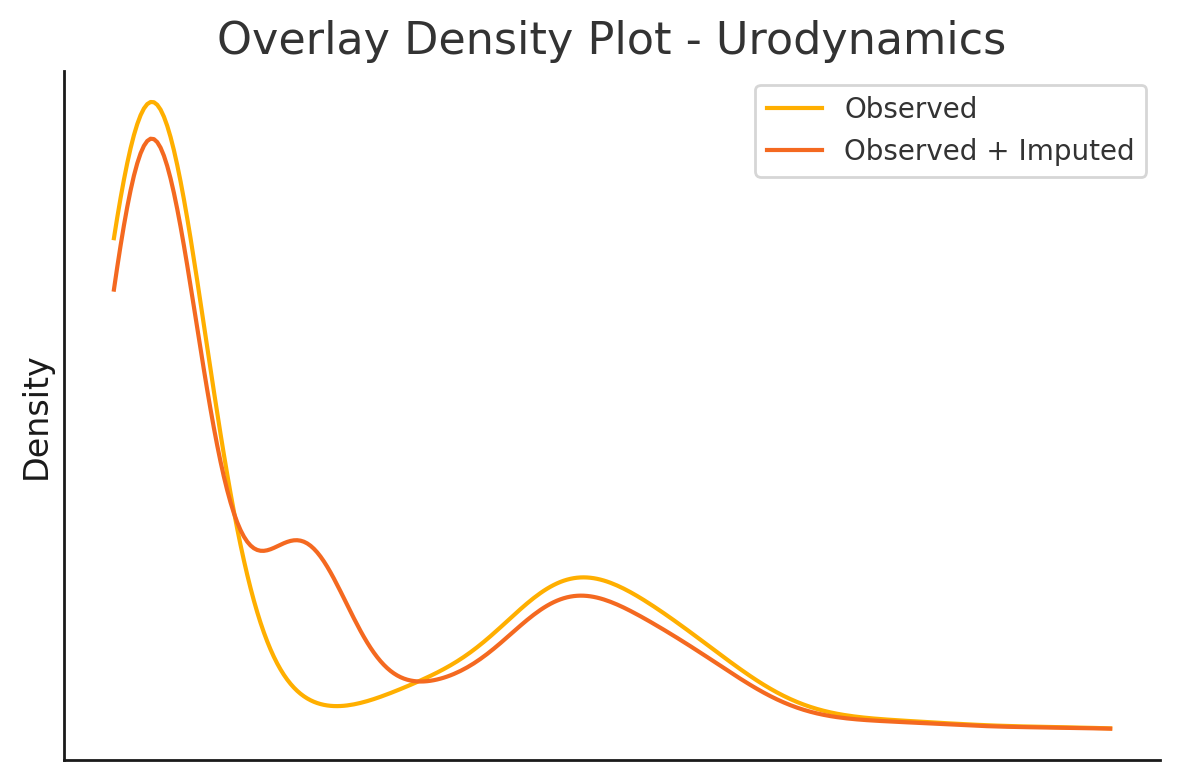

Supplement: Supplementary file 4 [file Image3.png]
